# Microwave-dried black soldier fly larvae enhance growth, intestinal health, and humoral immunity in broiler chickens: A functional feed approach

**DOI:** 10.14202/vetworld.2025.1840-1849

**Published:** 2025-07-08

**Authors:** Osfar Sjofjan, Ari Ardiantoro, Inaayah Novitri Cahyawati, Amalia Iffah Jihana, Muhammad Halim Natsir, Yuli Frita Nuningtyas, Danung Nur Adli

**Affiliations:** 1 Department of Feed and Animal Nutrition, Faculty of Animal Science, Universitas Brawijaya, Malang, East Java, Indonesia; 2 Laboratory of Biotechnology, Faculty of Animal Science, Universitas Brawijaya, Malang, East Java, Indonesia; 3 Department of Feed and Animal Nutrition, Smart Livestock Industry Study Programme, Faculty of Animal Science, Universitas Brawijaya, Malang, East Java, Indonesia

**Keywords:** black soldier fly, broiler chickens, functional feed, humoral immunity, insulin-like growth factor-1, intestinal morphology, lauric acid, microwave drying

## Abstract

**Background and Aim:**

The global demand for sustainable animal protein sources has led to the exploration of insects as alternative feed ingredients. Among these, black soldier fly (BSF) larvae (*Hermetia illucens*) have demonstrated significant nutritional and functional potential. This study investigated the effects of microwave-dried BSF larvae meal (MDBSFM) on growth performance, intestinal morphology, humoral immune response, and insulin-like growth factor-1 (IGF-1) messenger RNA (mRNA) expression in broiler chickens.

**Materials and Methods:**

A total of 160 unsexed Lohmann broiler chickens were randomly allocated to five dietary treatments (T0–T4), with MDBSFM supplementation levels of 0%, 0.25%, 0.50%, 0.75%, and 1.00%, respectively. Each treatment consisted of four replicates, each with eight birds. Growth performance metrics, organ weights, intestinal histomorphology, immunoglobulin levels (IgA and IgY), and IGF-1 mRNA expression were measured. Fatty acid composition of MDBSFM was analyzed by gas chromatography.

**Results:**

MDBSFM supplementation significantly improved body weight gain, final body weight, and feed conversion ratio (p < 0.05), with optimal outcomes observed at 0.75% inclusion. Villus height in the ileum was markedly increased in the 0.50% and 0.75% groups (p < 0.01), suggesting enhanced nutrient absorption. IgA and IgY concentrations were significantly elevated in response to MDBSFM (p < 0.05), particularly in the T3 and T4 groups. Although IGF-1 mRNA expression did not differ significantly (p = 0.58), the highest fold change (1.54) was noted in the 1.00% group.

**Conclusion:**

Microwave-dried BSF larvae meal represents a promising functional feed additive capable of improving growth, intestinal health, and humoral immune responses in broilers without adverse effects. The high lauric acid content and bioactive compounds in MDBSFM may contribute to these beneficial effects. While IGF-1 upregulation trends warrant further molecular investigation, MDBSFM offers a viable alternative to antibiotic growth promoters in poultry diets. Future research should focus on microbiota profiling and large-scale commercial validation.

## INTRODUCTION

The rapid increase in global population, combined with mounting concerns over food, feed, and energy security, environmental degradation due to climate change, and a rising demand for animal protein from the expanding middle class, has intensified the search for alternative and sustainable food and feed sources [1]. Insects have emerged as a promising and ecologically sound solution. Unlike conventional protein sources such as red and white meats, insects can be reared on waste or underutilized organic substrates, thereby reducing environmental burdens and enha-ncing circular resource use [2]. Insect farming generates substantially lower greenhouse gas and ammonia emi-ssions than both ruminant and nonruminant livestock systems and requires significantly less water than traditional ruminant farming, positioning it as a more environmentally sustainable alternative [3].

Beyond ecological advantages, insects offer notable nutritional benefits, being rich in protein, fiber, essential minerals, and other bioactive compounds [4]. Entomophagy, the consumption of insects, has also been associated with socioeconomic gains. For inst-ance, insect farming has been shown to alleviate food insecurity and support livelihoods in low-income reg-ions, particularly in Africa [5]. Globally, more than two billion people traditionally consume insects as part of their diets, reflecting long-standing cultural and nutr-itional practices [5]. Among the wide range of edible insect species, black soldier fly (BSF) larvae (*Hermetia illucens*) have received growing attention for their sustainability and functional value as animal feed. BSF larvae are particularly appreciated for their high-quality nutrient profile and documented benefits to animal growth and health [6, 7]. Prior studies by Schiavone *et al*. [4], FAO [5], and Dabbou *et al*. [6] have primarily evaluated BSF larvae as a feed additive in broiler chicken diets, where their inclusion has been associated with improved growth performance, enhanced nutrient utilization, and better feed efficiency.

Despite the growing body of evidence supporting the use of BSF larvae as an alternative protein source in broiler nutrition, substantial gaps remain concer-ning the specific processing methods that may influ-ence their bioactivity and physiological impact. Most existing studies have evaluated oven-dried or freeze-dried BSF larvae, with limited exploration of micro-wave drying, a technique that may better preserve heat-sensitive nutrients, such as medium-chain fatty acids (MCFAs) and bioactive peptides. Moreover, the majority of previous research has primarily focused on performance metrics such as body weight gain (BWG) and feed conversion ratio (FCR), with minimal attention to functional outcomes related to intestinal health, immune modulation, and endocrine regulation. Specifically, there is a lack of studies investigating the effects of microwave-dried BSF larvae meal (MDBSFM) on the expression of insulin-like growth factor-1 (IGF-1), a key regulator of muscle development and metabolic function in broiler chickens. In addition, few studies have quantified humoral immune responses, such as immunoglobulin A (IgA) and immunoglobulin Y (IgY) levels, following MDBSFM supplementation, nor have they examined histomorphological changes in the gastrointestinal tract that underlie enhanced nutrient absorption. Importantly, no published studies to date have simultaneously evaluated growth performance, intestinal morphology, humoral immunity, and *IGF-1* gene expression in broilers fed MDBSFM, representing a critical knowledge gap in the functional assessment of this emerging feed ingredient.

This study aimed to evaluate the nutritional and functional effects of dietary inclusion of MDBSFM on fast-growing broiler chickens. Specifically, it assessed the influence of graded levels of MDBSFM (0.25%–1.00%) on growth performance parameters (BWG and FCR), intestinal histomorphology (villus height [VH] and crypt depth [CD]), humoral immune responses (serum IgA and IgY concentrations), and IGF-1 messenger RNA (mRNA) expression using quantitative real-time polymerase chain reaction (qRT-PCR). By integrating phenotypic, immunological, and molecular endpoints, this research aimed to elucidate the potential of MDBSFM as both a sustainable protein source and a functional feed additive that can enhance immune competence and growth-related gene expression in poultry production systems. This multidimensional approach contributes to the evidence base for the strategic use of insect-derived feed ingredients in antibiotic-free broiler diets.

## MATERIALS AND METHODS

### Study area and ethical approval

Ethical approval was obtained from the Health Research Ethics Committee of the Faculty of Medicine, Universitas Brawijaya (Ref: 139/EC/KEPK/06/2023), granted on June 27, 2023. The ethical review process adhered to the Declaration of Helsinki and the institu-tional research protocol.

### Study period and location

The study was conducted from June 5^th^ to July 10^th^, 2023 at the Sumber Sekar Poultry Research Station, Dau subdistrict, Malang Regency, East Java Province, Indonesia (latitude 7.9185° S and longitude 112.5757° E). Serum biochemical analyses were conc-urrently performed at Satwa Sehat Laboratory, Malang City, East Java Province, Indonesia (latitude 7.9549° S and longitude 112.5947° E), while the mRNA expression analysis was conducted at the Biotechnology Labor-atory, Faculty of Animal Science, Universitas Brawijaya, Malang City.

### Preparation of microwave-dried BSF larvae

BSFs were obtained from PT. Diola Karya (Larvioola, Indonesia), Malang. Eggs were incubated for 3 days, and 1-day-old larvae were harvested and reared in media-specific treatments: Fruit waste (T0), food waste (T1), tofu byproduct (T2), 50% T0 + 50% T2 (T3), and 50% T0 + 50% T1 (T4) (Table 1). Waste materials were sourced locally from markets, restaurants, households, and tofu factories. After weighing 2000 larvae (0.06 g ± 0.04/20 larvae pool), they were randomly divided into ten groups and reared in ponds (64 × 34 × 10 cm) under controlled conditions (29°C, 78% relative humidity, 18L:6D photoperiod). Larvae were fasted for 24 h to empty gut contents before drying. Microwave drying was performed using a Sharp oven (PT. Sharp Electronics, Indonesia) at 800 W for 14 min, with larvae spread in a single layer to ensure uniform exposure. These settings were selected based on preliminary trials to optimize drying efficiency and nutritional retention.

**Table 1:** Nutritional content of various maggot media.

Nutrient (%)	Media				

	Fruit waste	Food waste	Tofu by product	50% fruit waste and 50% tofu by product	50% fruit and 50% food waste
Dry matter	93.07	40.44	11.00	52.04	66.76
Ash	2.68	1.67	4.27	3.48	2.18
Crude protein	7.76	15.03	20.03	13.90	11.40
Fat	1.09	12.45	2.89	1.99	6.77
Crude fiber	6.03	19.77	2.48	4.26	12.90

Source: Central Laboratory, Universitas Muhammadiyah Malang (2023)

**Table 2:** Nutritional content of various maggot media.

SCFA	Media				

	T0	T1	T2	T3	T4
Butyric acid	0.35	7.84	<0.1	0.16	0.16
Lauric acid	34.28	35.14	35.99	37.56	38.39
Myristic acid	8.58	7.35	8.90	9.36	9.31
Palmitic acid	19.25	17.74	18.34	17.45	18.43
Heptadecanoic acid	2.57	2.03	2.84	2.82	2.91
Decanoic acid	1.37	1.42	1.31	1.23	1.31
Arachidic acid	10.41	8.98	10.11	10.22	8.80
Heneicosanoic acid	0.44	0.24	0.51	0.49	<0.1
Docosanoic acid	<0.1	0.51	<0.1	0.58	<0.1
Butyric acid	0.35	7.84	<0.1	0.16	0.16
Total	77.25	81.25	78.00	79.87	79.31
MUFA					
Palmitoleic acid	0.42	0.41	0.40	0.35	0.37
Oleic acid	1.84	1.64	1.69	1.91	1.72
Myristoleic acid	0.22	0.18	<0.1	<0.1	<0.1
Erucic acid	0.28	0.28	<0.1	0.31	0.41
Methyl nervonate	1.44	1.44	1.34	1.44	1.46
Total	4.20	3.95	3.43	4.01	3.96
PUFA					
Linoleic acid (C18:2 n-6)	16.86	13.51	16.68	14.42	15.26
Linolenic acid (C18:3 n-3)	0.91	0.70	0.89	1.03	0.65
Docosahexaenoic acid (DHA)	0.76	0.59	1.00	0.65	0.83
Eicosapentaenoic acid (EPA)	<0.1	<0.1	<0.1	<0.1	<0.1
Total	18.53	14.80	18.57	16.10	16.74

SFA=Short-chain fatty acids, MUFA=Monounsaturated fatty acid, PUFA=Polyunsaturated fatty acids, T0=Fruit waste, T1=Food waste, T2=Tofu byproduct, T3=A combination of 50% T0 and 50% tofu byproduct; and T4=A combination of 50% T0 and 50% food waste. Source: Laboratory of Penelitian dan Pengujian Terpadu (LPPT), Universitas Gadjah Mada, Yogyakarta, (2023)

### Experimental design and broiler management

A total of 160 unsexed MB-Lohmann broiler chickens were obtained from PT. Japfa Comfeed Tbk (East Java, Indonesia) and randomly assigned to five dietary treatments (T0–T4), each with four replicates of eight birds. A completely randomized design was implemented. Birds were housed in pens (1 × 1 × 2 m) under progressive lighting conditions: 23L:1D in week 1, 20L:4D in week 2, and 18L:6D thereafter. Brooding temperature started at 32°C and was reduced weekly to 25°C. Birds received *ad libitum* access to feed and water and were vaccinated against Newcastle disease (ND) and infectious bronchitis using inactivated ND La Sota strain and Massachusetts M-41 with oil adjuvant and thiomersal.

### Diet formulation and feeding

The basal diet was composed of plant-based ingredients, including maize, cassava meal, rice bran, palm kernel meal, crude palm oil, salt, DL-methionine, and a vitamin-mineral premix (Table 3). Diets were formulated to be isoenergetic and isonitrogenous: Starter diet (3300 kcal/kg metabolizable energy [ME], 20% crude protein [CP]) and finisher diet (3100 kcal/kg ME, 19% CP). MDBSFM was added at 0% (T0), 0.25% (T1), 0.50% (T2), 0.75% (T3), and 1.00% (T4) on top of the base diet. Formulations met or exceeded NRC (1994) nutrient requirements. The exclusion of anti-biotic growth promoters (AGPs) was in line with PERMENTAN/14/16/2017.

**Table 3:** Feed formulation and nutrient composition during experiments.

Ingredients	Starter (1–21 days) (%)	Finisher (22–35 days) (%)
Maize	53.00	50.00
Soya bean meal	25.00	23.00
DDGS	10.00	10.00
Rice bran	5.00	10.00
Cassava meal	5.50	5.00
Salt	0.15	0.15
Crude palm oil	1.00	1.00
Commercial enzyme	0.20	0.20
Phytase acid	0.50	0.50
Vitamin premix	0.20	0.20
Amino acid	0.15	0.15
Total	100.70	100.20
Calculated composition (%)		
Dry matter	88.00	88.00
Crude protein	20.00	19.00
Fat	5.00	5.00
Crude fiber	5.00	5.00
Ash	8.00	8.00
Metabolizable energy (Kcal/kg)	3300.00	3100
Calcium	1.10	1.10
Phosphorus	0.60	0.60
Lysine	1.20	1.20
Methionine	0.45	0.45
Proximate composition		
Dry matter	92.50	92.54
Crude protein	22.88	19.13
Crude fiber	3.24	2.95
Fat	5.04	7.55
Ash	6.97	11.07
Metabolizable energy (Kcal/kg)	3104.55	3100.44

d=Day, DDGS=Dried distillers’ soluble grains; kcal, kilocalorie, kg=Kilogram, Source: Feed and Animal Nutrition Laboratory, Universitas Brawijaya, Malang (2023)

### Fatty acid profile measurements

Fatty acids were extracted from MDBSFM using the Soxhlet method, followed by methylation using 0.5 N NaOH in methanol and 20% BF_3_ at 80°C. Samples were cooled, treated with NaCl and isooctane, and filtered through anhydrous Na_2_SO_4_. The methyl est-ers were analyzed by gas chromatography (Shimadzu GC-2010, Guangdong, China) using a cyanopropyl methyl sil column (60 m × 0.25 mm × 0.25 μm). The flame ionization detector and injector were set at 240°C and 220°C, respectively, with a temperature program of 125°C–225°C. Peak identification was based on the comparison of retention time with known standards. The external standard method was used to calculate fatty acid concentrations.

### Growth performance measurements

Initial body weight (IBW) was recorded on the 1^st^ day. Weekly measurements of BWG, final body weight (FBW), feed intake (FI), and FCR were recorded per pen on days 7, 14, 21, 28, and 35. Mortality and abnormal conditions (e.g., lameness, lethargy, and ruffled feathers) were documented daily. FCR values were corrected for mortality. Average daily gain (ADG) and average daily FI (ADFI) were computed.

### Organ weights and intestinal measurements

On day 35, one bird per replicate was randomly selected. After live weight recording and euthanasia through exsanguination (jugular vein, trachea, and carotid artery), internal organs – liver, heart, gizzard, abdominal fat, and lymphoid tissues – were excised and weighed. Small intestine length was also measured.

### Blood collection and biochemical analysis

Broilers were fasted for 16 h before blood sampling (17:00–19:00). One milliliter of blood was collected from the wing vein (Vena brachialis) using a 23G × 1¼ syringe. Samples were placed in lithium heparin-coated microtubes and centrifuged at 704.34 × *g* for 15 min (Hettich^®^ Universal 320/320R centrifuge, Andreas Hett-ich GmbH, Germany). Plasma was stored at −20°C. Parameters analyzed included hemoglobin, packed cell volume, hematocrit, erythrocytes, leukocytes, mean corpuscular volume, mean corpuscular hemoglobin, mean corpuscular hemoglobin concentration, platelets (PLTs), glucose, cholesterol, and low-density lipop-rotein, using a Mindray BC-2800Vet hematology anal-yzer (Nanshan, Shenzhen, China).

### Intestinal histomorphology

Intestinal samples (0.5–1 cm) were fixed, embedded in paraffin, and sectioned at a thickness of 5 μm. Slides were stained with hematoxylin and eosin (Sigma–Aldrich, Merck KGaA, based in Darmstadt, Germany) and mounted with Entellan. Observations were performed under a Nikon Eclipse Ei microscope (Tokyo, Japan) (100×), and images were captured using an Optilab camera (Yogyakarta, Indonesia). VH, CD, and width were analyzed using Image Raster software Version 3.7 (Sleman, Yogyakarta, Indonesia).

### RNA extraction and gene expression analysis

Post-euthanasia, pectoral muscle, liver, gallbla-dder, brain, and spleen samples were collected, deco-ntaminated with 70% alcohol, and preserved using DNA/RNA shield. Samples were stored at −20°C in a Bio-Base Freezer (Jinan, Shandong, China). RNA extraction was performed using the Quick-RNA™ MiniPrep Plus kit (Zymo Research, California, USA) and purity was verified by NanoDrop spectrophotometry (A260/A280 ratio 1.8–2.0; Thermo Fisher Scientific, Wilmington, DE, USA). RNA integrity was confirmed by electrophoresis on a 1.5% agarose gel. Reverse transcription was carried out using 2 μL of 5× RT master mix II. Synthesized cDNA was used for qRT-PCR with SYBR Green Supermix (Bio-Rad Laboratories Inc., Hercules, California, USA) on a CFX OPUS 96 system (Bio-Rad Laboratories Inc.) IGF-1 and GAPDH primers (Bio-Rad Laboratories Inc.) were designed using MEGA software v11.0.13 (Pennsylvania, USA) based on GenBank (NM_001004384.3) (Table 4). Primer efficiency ranged from 96% to 103%, and all reactions were conducted in technical triplicate with melt curve analysis to verify specificity.

**Table 4:** Gene primer sequence.

Name	Gene number	Strand	Sequence (5’–3’)	Size (bp)	Annealing temperature (°C)
IGF-1	NM_001004384.3	Forward	GTTCGTATGTGGAGACAGAG	143	62
		Reverse	CTGGAGATGTACTGTGCTC		

IGF-1=Insulin-like growth factor 1, °C=Degree of elasticity, NM=Number

### Humoral immune response evaluation

Plasma samples were dilapidated and centrifuged at 10,000 × *g* for 15 min at 4°C. IgY was precipitated from 0.5 mL of supernatant, incubated for 24 h, centrifuged again, and reconstituted in PBS. IgA was measured from jejunal mucosa and serum using a chicken IgA enzyme-linked immunosorbent assay (ELISA) kit (RK00583, ABclonal, Woburn, MA); IgY was quantified using a commercial chicken IgY ELISA kit (ab157693: Abcam plc. Cambridge, UK). ELISA plates were developed using horseradish peroxidase-conjugated antibodies and 3,3′,5,5′-Tetramethylbenzidine substrate. OD was measured at 450 nm using a Labtron LMPR-A12 reader (Labtron Equipment Ltd., Camberley, Surrey, UK). Concentrations were determined through standard curve interpolation.

### Statistical analysis

The analysis and coding of all the data were executed through R software vers-ion 4.3.3 (2024--01--28 UCRT: R Foundation for Stati-stical Computing, Vienna, Austria) – “Ocean Storm” x86_64--W64--mingw32/X64 computing, incorporating the libraries “readxl;” “xlsx;” “dplyr;” “multicompView;” and “nlme.” For data analysis, a general linear model (GLM) approach was applied through one-way analysis of variance (ANOVA) to evaluate the fixed effects of dietary treatments (T0–T4) on the response variables, including growth performance, organ weights, intestinal histomorphology, blood biochemical parameters, imm-une responses (IgA and IgY), and *IGF-1* gene expression levels. Tukey’s Honestly Significant Difference (HSD) test was used for *post hoc* pairwise comparisons where ANOVA indicated significance (p < 0.05). All values are presented as the mean ± standard deviations (SDs). The modeling approach used was as follows:

 Y_ijk_ = μ + τ_j_ + e_ijk_(1)


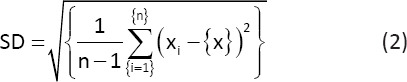


where (1) Y_ijk_ = The observed value of the dependent variable for the k-th observation in the j-th treatment group, μ = The overall mean value, τ_j_ = The fixed effect of the j-th dietary treatment (T0-T4), and e_ijk_= The residual value from unpredictable error. (2) SD = Standard deviation, X_i_ = Each individual observation, n = Number of observations, X = Sample mean. The significance of the treatment effects was evaluated using one-way analysis of variance. The results were considered statistically significant when p < 0.05 and trended toward significance when 0.05 ≤ p < 0.10. Tukey’s HSD test was used to compare the treatments as appropriate. T0: Basal diet; T1: Basal diet + 0.25% MDBSFM; T2: Basal diet + 0.50% MDBSFM; T3: Basal diet + 0.75% MDBSFM. The quantitative expression number was calculated as follows:

 {Fold Change}= 2^{-{ΔΔC_t}}^ (3)

C_t_ = Threshold cycle value (number of cycles needed to reach a defined fluorescence threshold), ΔC_t_, target −C_t_, reference = Difference between the target gene and the reference (housekeeping) gene,

ΔΔC_t_ = ΔC_t_, sample - ΔC_t_, control = Difference between the sample and control groups. Fold change >1: Upregulation of gene expression; fold change <1: Downregulation of gene expression; fold change = 1: No change in expression.

## RESULTS

### Fatty acid composition of MDBSFM

The fatty acid profile of MDBSFM varied depend-ing on the larval substrate (Table 2). Saturated fatty acids (SFAs) were the predominant class in all treatments, ranging from 77.25% in T0 to 81.25% in T1. Lauric acid (C12:0) was the most abundant SFA, increasing progressively from 34.28% in T0 to 38.39% in T4 (50:50 fruit and T1). A notably high concentration of butyric acid (7.84%) was observed in T1, while it remained minimal in other treatments (<0.1%–0.35%). Myristic, palmitic, and arachidic acids showed moderate but consistent variation across treatments.

Monounsaturated fatty acids were present in lower proportions, ranging from 3.43% in T2 to 4.20% in T0, with oleic acid and methyl nervonate being the most prominent. Polyunsaturated fatty acids (PUFAs) were the second-largest category, ranging from 14.80% in T1 to 18.57% in T2. Linoleic acid (C18:2 n-6) was the major PUFA, followed by linolenic acid (C18:3 n-3) and docosahexaenoic acid.

### Growth performance and carcass traits

Broiler chickens fed MDBSFM-supplemented diets showed significant improvements in growth perfor-mance, particularly in the 0.50% (T2) and 0.75% (T3) groups (Table 5). BWG and FBW were significantly increased (p < 0.05) compared to the control group. FCR showed a downward trend in the MDBSFM-fed birds (p < 0.01), although IBW and FI did not significantly differ (p > 0.05). Mortality rates remained low across all groups.

**Table 5:** Effects of MDBSFM on broiler chicken growth performance.

No.	Response variables	Unit	T0 (n = 4)	T1 (n = 4)	T2 (n = 4)	T3 (n = 4)	T4 (n = 4)	p-value	Note

			Growth performance						
1	IBW	(g/head)	45.00 ± 0.82	45.00 ± 0.82	44.50 ± 0.58	44.75 ± 1.50	45.75 ± 1.50	0.59	NS
2	BWG	(g/d/head)	7.06 ± 0.13^a^	7.25 ± 0.14^ab^	7.37 ± 0.19^c^	7.54 ± 0.17^d^	7.61 ± 0.07^e^	0.0004	S
3	FBW	(g/head)	2025.00 ± 35.72^a^	2076.00 ± 38.94^b^	2112.75 ± 52.97^c^	2158.75 ± 45.92^d^	2180.00 ± 19.58^e^	0.0004	S
4	FI	(g/d/head)	11.32 ± 0.09	11.38 ± 0.09	11.44 ± 0.06	11.46 ± 0.06	11.49 ± 0.06	0.254	NS
5	FCR	-	1.60 ± 0.02^a^	1.57 ± 0.03^b^	1.55 ± 0.04^c^	1.51 ± 0.04^d^	1.50 ± 0.01^e^	0.001	S
6	Mortality	(%)	0.50 ± 0.00	0.25 ± 0.00	0.00 ± 0.00	0.00 ± 0.00	0.00 ± 0.00	0.54	S

BWG=Body weight gain, CON = Control, d=Day, FCR=Feed conversion ratio, FI=Feed intake, FBW=Final body weight, g=Gram, IBW=Initial body weight, NS=Not significant, n = Number of birds as sample, S=Significant, TRT=Treatments

There were no statistically significant differences in organ weights or intestinal lengths (Table 6; p > 0.05). However, numerically reduced liver and abdominal fat weights were observed in the 0.25% and 0.50% MDBSFM groups, suggesting a trend toward leaner carcass composition. Similar numerical trends were observed in the heart and lymphoid organs.

**Table 6:** Effects of MDBSFM on the organ and intestinal weight of broiler chickens.

No.	Response variables	Unit	T0 (n = 4)	T1 ( n = 4)	T2 (n = 4)	T3 ( n = 4)	T4 ( n = 4)	p-value	Note

			Organ weight						
1	Liver	(g)	44.52 ± 7.65	41.95 ± 7.04	39.97 ± 4.92	38.92 ± 2.97	42.33 ± 4.09	0.66	NS
2	Heart	(g)	9.20 ± 2.67	10.47 ± 0.78	10.27 ± 1.45	9.70 ± 0.54	8.95 ± 1.37	0.58	NS
3	Lymph	(g)	2.42 ± 0.71	2.82 ± 0.62	2.10 ± 0.42	2.37 ± 0.79	2.55 ± 0.54	0.60	NS
4	Gizzard	(g)	28.80 ± 4.58	27.70 ± 2.86	26.15 ± 0.62	28.45 ± 3/76	28.80 ± 0.42	0.75	NS
5	Abdominal fat	(g)	37.55 ± 15.95	33.85 ± 4.53	33.85 ± 8.65	38.02 ± 7.98	39.82 ± 11.69	0.89	NS
6	Liver	(%)	2.18 ± 0.40	2.13 ± 0.36	1.95 ± 0.24	1.78 ± 0.08	2.17 ± 0.21	0.24	NS
7	Heart	(%)	0.45 ± 0.13	0.53 ± 0.04	0.50 ± 0.06	0.44 ± 0.03	0.45 ± 0.07	0.43	NS
8	Lymph	(%)	0.11 ± 0.03	0.13 ± 0.03	0.09 ± 0.02	0.10 ± 0.04	0.12 ± 0.03	0.43	NS
9	Gizzard	(%)	1.41 ± 0.21	1.40 ± 0.16	1.27 ± 0.01	1.31 ± 0.21	1.41 ± 0.10	0.60	NS
10	Abdominal fat	(%)	1.85 ± 0.84	1.71 ± 0.18	1.65 ± 0.42	1.84 ± 0.43	1.94 ± 0.56	0.93	NS

CON = Control, g=Gram, NS=Not significant, n = Number of birds included in the sample, TRT=Treatments, %=Percentage

### Ileal histomorphology

MDBSFM supplementation significantly incr-eased VH in the ileum, especially in T2 and T3 (T0: 265.32 ± 22.13 μm; T2: 400.94 ± 102.31 μm; T3: 401.69 ± 64.57 μm; p < 0.0001) (Table 7). Although CD, villus width, and VH: CD ratio did not differ significantly (p > 0.05), the positive trends observed support the hypothesis of enhanced absorptive capacity in MDBSFM-fed birds.

**Table 7:** Effects of MDBSFM on the intestinal histomorphology of broiler chickens.

No.	Response variables	Unit	T0 (n = 4)	T1 ( n = 4)	T2 ( n = 4)	T3 (n = 4)	T4 ( n = 4)	p-value	Note

			Intestinal histomorphology						
1	VH	(μm)	265.32 ± 22.13^a^	324.18 ± 81.40^b^	400.94 ± 102.31^c^	401.69 ± 64.57^c^	393.37 ± 80.37^b^	<.0001	S
2	CD	(μm)	84.49 ± 6.01	95.07 ± 33.51	126.76 ± 23.24	104.72 ± 7.74	121.42 ± 39.18	0.16	NS
3	VW	(μm)	1027.56 ± 83.80	1362.28 ± 376.40	1029.72 ± 147.38	1116.83 ± 217.23	1169.25 ± 367.92	0.405	NS
4	VH/CD ratio	-	3.17 ± 0.49	3.51 ± 0.62	3.14 ± 0.40	3.87 ± 0.85	3.43 ± 0.88	0.54	NS

CD=Crypt depth, CON = Control, VH=Villus height, VW=Villus width, L=Linear, TRT=Treatments, μm=micrometer

### Hematology and immune response

Most hematological parameters did not differ significantly across treatments (p > 0.05) (Table 8). However, PLT counts were significantly elevated in T1 and T2 (p = 0.003), suggesting a possible enhancement in hematopoietic or immune function. Erythrocyte and lymphocyte counts were also numerically higher in MDBSFM-fed birds compared to the control.

**Table 8:** Effects of MDBSFM on blood biochemical, gene expression, and humoral immune responses of broiler chickens.

No.	Response variables	Unit	T0 (n = 4)	T1 (n = 4)	T2 ( n = 4)	T3 (n = 4)	T4 ( n = 4)	p-value	Note

			Blood biochemical						
1	Hemoglobin	g/dL	15.20 ± 1.94	14.50 ± 1.49	13.17 ± 0.45	14.97 ± 1.97	15.47 ± 2.63	0.45	NS
2	Hematocrit	%	55.42 ± 7.61	56.27 ± 5.63	50.60 ± 1.70	55.72 ± 2.58	53.97 ± 3.04	0.45	NS
3	Erythrocytes	×10^6^/mm^3^	10.77 ± 0.42	9.87 ± 1.04	10.42 ± 0.83	9.65 ± 0.64	9.37 ± 0.39	0.07	NS
4	Leukocytes	×10^3^/mm^3^	19.27 ± 2.24	20.97 ± 2.53	21.45 ± 4.90	23.00 ± 5.98	25.02 ± 2.63	0.34	NS
5	MCV	fL	51.55 ± 7.77	57.10 ± 4.51	48.82 ± 5.38	57.85 ± 3.54	57.65 ± 1.25	0.06	NS
6	MCH	Pg	14.10 ± 1.34	15.02 ± 2.70	13.65 ± 1.04	14.37 ± 1.21	19.75 ± 3.13	0.14	NS
7	MCHC	%	27.67 ± 3.68	26.10 ± 4.92	26.05 ± 1.38	26.82 ± 2.71	28.821 ± 5.84	0.84	NS
8	PLT	×10^3^/mm^3^	646.27 ± 623.09^a^	1215.75 ± 195.39^b^	1207.50 ± 65.72^b^	894.30 ± 289.97^a^	1067.50 ± 292.91^ab^	0.003	S
9	Heterophils	%	35.35 ± 13.39	30.07 ± 6.59	40.45 ± 12.44	31.77 ± 11.72	27.25 ± 7.62	0.48	NS
		×10^9^/mm^3^	26.30 ± 15.45	26.30 ± 8.19	32.42 ± 23.14	24.17 ± 8.97	25.20 ± 8.68	0.92	NS
10	Lymphocytes	%	59.55 ± 18.13	68.87 ± 6.64	83.05 ± 14.46	79.57 ± 8.10	79.80 ± 12.48	0.09	NS
		×10^9^/mm^3^	210.80 ± 10.35	220.27 ± 14.23	212.48 ± 7.10	215.88 ± 15.90	222.10 ± 3.72	0.57	NS
11	Monocytes	%	5.15 ± 3.55	3.42 ± 0.91	2.40 ± 0.36	3.72 ± 1.08	3.20 ± 1.00	0.31	NS
		×10^9^/mm^3^	8.17 ± 3.16	8.85 ± 2.89	5.70 ± 0.94	9.55 ± 3.17	5.17 ± 2.79	0.14	NS

			**Humoral immune responses**						

12	IgA	(µg/mL)	2.55 ± 0.96^a^	3.92 ± 2.58^ab^	5.18 ± 1.14^b^	5.29 ± 2.14^b^	8.83 ± 3.10^c^	0.01	S
13	IgY	(mg/mL)	260.21 ± 67.89^a^	301.30 ± 92.01^ab^	346.83 ± 96.41^b^	348.94 ± 60.92^b^	315.71 ± 61.80 ^ab^	0.05	S

			**Gene expression**						

14	IGF-1	-	32.46 ± 1.04	33.67 ± 0.83	34.03 ± 1.26	34.32 ± 1.53	34.94 ± 1.35	0.11	NS
15	GAPDH (Housekeeping)	-	18.43 ± 0.38^a^	19.22 ± 0.37^ab^	19.43 ± 0.98^ab^	20.31 ± 1.72 ^ab^	21.24 ± 1.59^b^	0.03	NS
16	Fold Change	-	1.22 ± 0.99	0.92 ± 0.41^a^	1.04 ± 0.28^a^	1.20 ± 0.25^a^	1.54 ± 0.47^b^	0.58	NS

CON = Control, dl=Deciliter, fL=Femtoliter, g=Gram, IgA=Immunoglobulin A, IgY=Immunoglobulin Y, MCH=Mean corpuscular hemoglobin, MCHC=Mean corpuscular hemoglobin concentration, MCV=Mean corpuscular volume, mg=Milligram, mm=Millimeter, NS=Nonsignificant, n = Number of birds as sample, Pg=Pictograms, PLT=Platelet count, S=Significant, TRT=Treatments, µg=Micrograms

**Figure 1:**
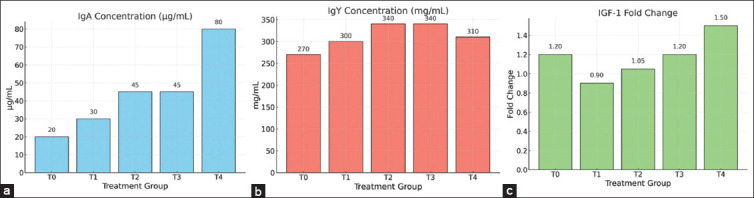
The effects of dietary supplementation with microwave-dried black soldier fly larvae meal (MDBSFM) on (a) immunoglobulin A (IgA), (b) immunoglobulin Y (IgY), and (c) insulin-like growth factor 1 (IGF-1) gene expression (fold change) in broiler chickens. Data are presented as mean values across treatment groups: T0 (control), T1 (0.25% MDBSFM), T2 (0.50% MDBSFM), T3 (0.75% MDBSFM), and T4 (1.00% MDBSFM). Supplementation with MDBSFM markedly increased IgA and IgY concentrations, with the highest values observed in T4. Although the IGF-1-fold change was not statistically significant, T4 cells also exhibited the greatest numerical increase, indicating the potential upregulation of gene expression.

Both IgA and IgY concentrations were signifi-cantly elevated in birds fed MDBSFM (Figure 1). IgA levels were significantly higher in T2, T3, and T4 (8.83 ± 3.10 μg/mL; p = 0.01), while IgY concentrations peaked in T3 (348.94 ± 60.92 mg/mL; p = 0.05), indicating improved humoral immune responses with MDBSFM inclusion.

### *IGF-1* gene expression

As shown in Table 8, MDBSFM supplementation numerically increased *IGF-1* gene expression across all treatment groups. Although the differences were not statistically significant (p = 0.58), all treated groups exhibited fold-change values greater than 1, indicating that the genes were upregulated. The T4 group showed the highest mean fold change (1.54), suggesting a potential biological effect (Figure 1). These findings imply that higher inclusion rates of MDBSFM, particularly at 1.00%, may enhance growth-related gene expression, warranting further investigation.

## DISCUSSION

### Effects of BSF larvae on broiler growth performance

According to Dabbou *et al*. [6], incorporating dietary BSF larvae and modified BSF did not adversely affect the growth performance of broiler chickens. Although significant trends were observed for live weight on day 33 (p = 0.096), ADFI (p = 0.062 and p = 0.074, respectively), and ADG (p = 0.080 and p = 0.095, respectively) during the grower-finisher and overall periods, the second treatment group demonstrated superior performance compared with other groups.

### Intestinal morphology and physiological health

Dabbou *et al*. [6] demonstrated that BSFL supple-mentation did not significantly affect the intestinal morphology of broiler chickens. The progression of morphometric indices across the intestinal sections, from the duodenum to the ileum, revealed maintained physiological development and nutrient absorption, indicating overall gut health in all groups, including the controls.

### Bioactive compounds in BSF and their functional roles

Insect-derived fats, particularly from BSF, are rich in MCFAs, notably lauric acid (C12:0), which pos-sesses antimicrobial and growth-promoting properties [8, 9]. These characteristics may enh-ance gut health and immune responses, ultimately contributing to improved growth performance and feed efficiency [6, 10]. BSF larvae fat also contains bioactive peptides that stimulate immunoglobulin production [11–16], enhancing poultry immune responses.

### Significance of lauric acid and thermal processing

Lauric acid, a saturated MCFA, remains relatively stable under thermal processing and exhibits antim-icrobial properties that improve gut health by reducing pathogenic bacteria and promoting favorable micro-biota [17, 18]. Microwave treatment enhances fat extraction by disrupting larval cellular structures and hydrolyzing triglycerides into free fatty acids, thereby increasing the availability of free lauric acid [19, 20]. While no evidence suggests that BSF larvae secrete additional lauric acid post-microwaving, the thermal process preserves or enhances the natural content and bioactivity of lauric acid and peptides [17, 20, 21].

### Impact on immune function

MDBSFM supplementation led to elevated concen-trations of IgA and IgY, particularly at higher inclusion rates (T3 and T4), likely due to the immunomodulatory effects of MCFAs and bioactive peptides in BSF larvae. Microwave drying may have facilitated the preservation and release of these compounds, thus enhancing mucosal and systemic immunity.

### Role of IGF-1 in growth and development

IGF-1 plays a vital role in skeletal muscle devel-opment, cell growth, differentiation, and metabolic regulation [22–26]. The IGF axis comprises growth hormone (GH), IGF-1, IGF-2, their receptors, and bin-ding proteins, collectively influencing metabolism, pro-liferation, and survival [27–30]. Previous research by Saxena *et al*. [31] and Chen *et al*. [32] links IGF-1 and IGF-2 expression to muscle growth, feed efficiency, and productivity in broiler chickens. MCFAs such as lauric acid can support IGF-1 synthesis by improving nutrient availability, reducing inflammation, and influencing hormonal regulation [33, 34].

### IGF-1 mRNA expression following MDBSFM feeding

The current findings revealed a trend toward IGF-1 mRNA upregulation in MDBSFM-fed broilers. Although not statistically significant, the increased fold-change values suggest a biologically meaningful effect, particularly at the 1.00% inclusion level. This supports the role of MDBSFM in stimulating muscle growth and metabolic regulation through the IGF pathway.

### Limitations and future directions

This experiment was conducted under controlled laboratory conditions with fast-growing broilers, which may not represent commercial production systems. Additionally, the lack of gut microbiota profiling lim-its our understanding of microbial-mediated immune modulation. Future research should include field trials and microbiome analyses to validate and expand upon these findings.

### Practical implications and sustainable applications

MDBSFM presents a sustainable and nutritionally functional alternative to conventional feed ingredients. Inclusion at 0.50%–1.00% improved growth performance and immune markers without the use of AGPs. Micro-wave drying maintains the functional integrity of key bioactive compounds. When produced on organic waste, BSF larvae farming supports circular economy principles. Nonetheless, comprehensive economic ana-lyses are necessary to assess scalability and regional feasibility in commercial poultry production.

## CONCLUSION

This study demonstrated that MDBSFM is a viable and functional feed additive for broiler chickens. The inclusion of MDBSFM at 0.50%–1.00% in the diet significantly enhanced growth performance, as evidenced by improved BWG, FCR, and FBW. Notably, the inclusion levels of 0.50% (T2) and 0.75% (T3) yielded the most pronounced effects on performance metrics. Histological analysis of the ileum revealed a significant increase in VH, indicating an enhanced nutrient absorptive capacity. Immunologically, MDBSFM supplementation led to marked elevations in serum IgA and IgY concentrations, particularly in the T2–T4 groups, reflecting enhanced humoral immunity. Although not statistically significant, all MDBSFM-treated groups exh-ibited numerical upregulation of IGF-1 mRNA expre-ssion, with the T4 group (1.00%) showing the highest fold-change, suggesting potential endocrine modulation related to growth regulation.

The primary strength of this study lies in its multidimensional evaluation of MDBSFM, integrating zootechnical, histomorphological, immunological, and molecular endpoints under controlled conditions. The use of microwave drying as a processing method offers practical advantages by preserving the bioactivity of MCFAs and peptides, such as lauric acid, which play critical roles in immune and metabolic regulation. In addition, the use of organic waste as a larval substrate underscores the sustainability of BSF production within a circular economy framework.

In conclusion, MDBSFM represents a promising, cost-effective, and environmentally sustainable feed ingredient that supports growth, gut integrity, and immune competence in broiler chickens without relying on AGPs. These findings provide a foundation for the broader adoption of insect-based feed ingre-dients in poultry production systems. However, further research is warranted to assess long-term effects under commercial field conditions, explore microbiota-mediated mechanisms, and evaluate economic feasib-ility across diverse production contexts.

## AUTHORS’ CONTRIBUTIONS

OS, MHN, and DNA: Conceptualized and supervised the study. AA, INC, AIJ, and DNA: Data curation, formal analysis, and drafted the manuscript. OS, MHN, and YFN: Conceptualized and supervised the study, visualization, and drafted and revised the manuscript. AA and DNA: Visualization. All authors have read and approved the final manuscript.

## References

[1] Fernando I, Siddiqui S.A, Nugraha W.S, Yudhistira B, Adli D.N, Nagdalian A.A, Blinov A.V, Mario M.B (2023). Overview of larvae of red palm weevil, *Rhynchophorus ferrugineus* (Olivier) (*Coleoptera*:*Curculionidae*), as human food. J. Insects Food Feed.

[2] Siddiqui S.A, Thanpandiyan K, Adli D.N, Yudhistira B, Fernando I, De Palo P (2025). Overview of the African palm weevil (*Rhynchophorus phoenicis*) as food and feed-a critical review. J. Insects Food Feed.

[3] Adli D.N (2021). Uses insects in poultry feed as replacement soya bean meal and fish meal in development countries:A systematic review. Livest. Res. Rur. Dev.

[4] Schiavone A, Cullere M, De Marco M, Meneguz M, Biasato I, Bergagna S, Dezzutto D, Gai F, Dabbou S, Gasco L, Dalle Zotte A (2016). Partial or total replacement of soybean oil by black soldier fly larvae (*H. illucens* L.) fat in broiler diets:Effect on growth performances, feed-choice, blood traits, carcass characteristics and meat quality. Ital. J. Anim. Sci.

[5] FAO (2023). The State of Food Security and Nutrition in the World 2023:Urbanization, Agrifood Systems Transformation and Healthy Diets Across the Rural-Urban Continuum. FAO, Italy, Rome..

[6] Dabbou S, Lauwaerts A, Ferrocino I, Biasato I, Sirri F, Zampiga M, Bergagna S, Pagliasso G, Gariglio M, Colombino E, Garcés Narro C, Gai F, Capucchio M.T, Gasco L, Cocolin L, Schiavone A (2021). Modified black soldier fly larva fat in broiler diet:Effects on performance, carcass traits, blood parameters, histomorphological features and gut microbiota. Animals.

[7] National Research Council (NRC) (1994). Nutrient Requirements of Poultry.Ninth Revised Edition.

[8] Makkar H.P, Tran G, Heuzé V, Ankers P (2014). State-of-the-art on use of insects as animal feed. Anim. Feed Sci. Tech.

[9] Phaengphairee P, Boontiam W, Wealleans A, Hong J, Kim Y.Y (2023). Dietary supplementation with full-fat *Hermetia illucens* larvae and multi-probiotics, as a substitute for antibiotics, improves the growth performance, gut health, and antioxidative capacity of weaned pigs. BMC Vet. Res..

[10] Attia Y.A, Bovera F, Asiry K.A, Alqurashi S, Alrefaei M.S (2023). Fish and black soldier fly meals as partial replacements for soybean meal can affect sustainability of productive performance, blood constituents, gut microbiota, and nutrient excretion of broiler chickens. Animals.

[11] Wong F.C, Lee Y.H, Ong J.H, Manan F.A, Sabri M.Z, Chai T.T (2023). Exploring the potential of black soldier Fly larval proteins as bioactive peptide sources through *in silico* gastrointestinal proteolysis:A cheminformatic investigation. Catalysts.

[12] Lu J, Guo Y, Muhmood A, Zeng B, Qiu Y, Wang P, Ren L (2022). Probing the antioxidant activity of functional proteins and bioactive peptides in *Hermetia illucens* larvae fed with food wastes. Sci. Rep..

[13] Leni G, Del Vecchio L, Dellapina C, Moliterni V.M, Caligiani A, Cirlini M (2024). Black soldier fly larvae grown on hemp fiber:Nutritional composition and production of potential bioactive peptides. Macromol.

[14] Zhu D, Huang X, Tu F, Wang C, Yang F (2020). Preparation, antioxidant activity evaluation, and identification of antioxidant peptide from black soldier fly (*Hermetia illucens L.*) larvae. J. Food Biochem.

[15] Pimchan T, Hamzeh A, Siringan P, Thumanu K, Hanboonsong Y, Yongsawatdigul J (2024). Antibacterial peptides from black soldier fly (*Hermetia illucens*) larvae:Mode of action and characterization. Sci. Rep..

[16] Meng Y, Zhang X, Zhang Z, Li J, Zheng P, Li J, Xu J, Xian J, Lu Y (2023). Effects of microorganisms on growth performance, body composition, digestive enzyme activity, intestinal bacteria flora and antimicrobial peptide (AMP) content of black soldier fly larvae (*Hermetia illucens*). Animals.

[17] Jayadas N.H, Nair K.P (2006). Coconut oil as base oil for industrial lubricants-evaluation and modification of thermal, oxidative and low temperature properties. Tribol. Int.

[18] Inzaghi E, Pampanini V, Deodati A, Cianfarani S (2022). The effects of nutrition on linear growth. Nutrients.

[19] Deruytter D, Gasco L, Gligorescu A, Yakti W, Coudron C.L, Meneguz M, Grosso F, Shumo M, Frooninckx L, Noyens I, Bellezza Oddon S, Biasato I, Spranghers T, Oonincx D.G, Vandenberg G, Bosch G (2022). The (im)poss-ible standardisation of insect feed experiments:A BSF case study. J. Insects Food Feed.

[20] Saifuddin N, Refal H, Kumaran P (2014). Rapid purification of glycerol by-product from biodiesel production through combined process of microwave assisted acidification and adsorption via chitosan immobilized with yeast. Res. J. Appl. Sci. Eng. Technol.

[21] Rodríguez-Alcalá L.M, Alonso L, Fontecha J (2014). Stability of fatty acid composition after thermal, high pressure, and microwave processing of cow milk as affected by polyunsaturated fatty acid concentration. J. Dairy Sci.

[22] Nery J, Gasco L, Dabbou S, Schiavone A (2018). Protein composition and digestibility of black soldier fly larvae in broiler chickens revisited according to the recent nitrogen-protein conversion ratio. J. Insects Food Feed.

[23] Nakagawa A, Sakamoto T, Kanost M.R, Tabunoki H (2023). The development of new methods to stimulate the production of antimicrobial peptides in the larvae of the black soldier fly *Hermetia illucens*. Int. J. Mol. Sci..

[24] Guo Y, Zhang K, Geng W, Chen B, Wang D, Wang Z, Tian W, Li H, Zhang Y, Jiang R, Li Z, Tian Y, Kang X, Liu X (2023). Evolutionary analysis and functional characterization reveal the role of the insulin-like growth factor system in a diversified selection of chickens (*Gallus gallus*). Poult. Sci..

[25] Kadlec J, Hosnedlová B, Rehout V, Vecerek L, Hanusová L (2011). *Insulin-like growth factor-I* gene polymorphism association with growth, and slaughter characteristics in broiler chickens. J. Agrobiol..

[26] Wang W.J, Guo Y.Q, Xie K.J, Li Y.D, Li Z.W, Wang N, Xiao F, Guo H.S, Li H, Wang S.Z (2021). A functional variant in the promoter region of *IGF1* gene is associated with chicken abdominal fat deposition. Domest. Anim. Endocrinol..

[27] LeRoith D, Holly J.M, Forbes B.E (2021). Insulin-like growth factors:Ligands, binding proteins, and receptors. Mol. Metabol..

[28] Pfaffl M.W, Georgieva T.M, Georgiev I.P, Ontsouka E, Hageleit M, Blum J.W (2002). Real-time RT-PCR quantification of insulin-like growth factor (IGF)-1, IGF-1 receptor, IGF-2, IGF-2 receptor, insulin receptor, growth hormone receptor, IGF-binding proteins 1, 2 and 3 in the bovine species. Domest. Anim. Endocrinol.

[29] DeBerardinis R.J, Lum J.J, Hatzivassiliou G, Thompson C.B (2008). The biology of cancer:Metabolic reprogramming fuels cell growth and proliferation. Cell. Metab.

[30] Fritz V, Fajas L (2010). Metabolism and proliferation share common regulatory pathways in cancer cells. Oncogenes.

[31] Saxena R, Saxena V.K, Tripathi V, Mir N.A, Dev K, Begum J, Agarwal R, Goel A (2020). Dynamics of gene expression of hormones involved in the growth of broiler chickens in response to the dietary protein and energy changes. Gen. Comp. Endocrinol..

[32] Chen P, Li S, Zhou Z, Wang X, Shi D, Li Z, Xiao Y (2022). Liver fat metabolism of broilers regulated by *Bacillus amyloliquefaciens* TL by stimulating IGF-1 secretion and regulating the IGF signalling pathway. Front. Microbiol..

[33] Gomez-Osorio L.M, Yepes-Medina V, Ballou A, Parini M, Angel R (2021). Short and medium chain fatty acids and their derivatives as a natural strategy in the control of necrotic enteritis and microbial homeostasis in broiler chickens. Front. Vet. Sci..

[34] Kareem K.Y, Loh T.C, Foo H.L, Akit H, Samsudin A.A (2016). Effects of dietary postbiotic and inulin on growth performance, IGF1 and GHR mRNA expression, faecal microbiota and volatile fatty acids in broilers. BMC Vet. Res..

